# Mitochondria-associated regulation in adipose tissues and potential reagents for obesity intervention

**DOI:** 10.3389/fendo.2023.1132342

**Published:** 2023-06-16

**Authors:** Yali Zheng, Ni Yang, Yueshan Pang, Yanju Gong, Hong Yang, Weijun Ding, Hongya Yang

**Affiliations:** ^1^ Department of Fundamental Medicine, Chengdu University of Traditional Chinese Medicine, Chengdu, China; ^2^ School of Medical and Life Sciences/Reproductive & Women-Children Hospital, Chengdu University of Traditional Chinese Medicine, Chengdu, China

**Keywords:** obesity, mitochondria, white adipose tissue (WAT), brown adipose tissue (BAT), UCP1

## Abstract

**Introduction:**

A systematic review analysis was used to assess the profile of mitochondrial involvement in adipose tissue regulation and potential reagents to intervene in obesity through the mitochondrial pathway.

**Methods:**

Three databases, PubMed, Web of Science, and Embase, were searched online for literature associated with mitochondria, obesity, white adipose tissue, and brown adipose tissue published from the time of their creation until June 22, 2022, and each paper was screened.

**Results:**

568 papers were identified, of which 134 papers met the initial selection criteria, 76 were selected after full-text review, and 6 were identified after additional searches. A full-text review of the included 82 papers was performed.

**Conclusion:**

Mitochondria play a key role in adipose tissue metabolism and energy homeostasis, including as potential therapeutic agents for obesity.

## 1 Introduction

Adipose tissue, as a major energy storage site, is involved in physiological regulation of the whole body, including energy maintenance, insulin sensitivity, and food intake. Adipose tissue includes white adipose tissue (WAT), brown adipose tissue (BAT), and beige adipose tissue. Distinct fat depots have very unique biochemical properties and association with metabolic diseases (1). WAT stores excess energy as triglycerides (TG) and secretes adipokines such as leptin, TNF-α, and lipocalin, which contribute to the regulation of energy homeostasis (2), white adipocytes consist of single-compartment lipid droplets. BAT and beige adipose tissue are characterized by multi-compartment lipid droplets, high mitochondrial density and expression of uncoupling protein 1 (UCP1), and BAT consumes energy through non-fibrillatory thermogenesis by mitochondria (3). Adipose tissue is highly plastic and its restriction can drive pathological consequences such as obesity and metabolic diseases. As a risk factor for several metabolic diseases, obesity contributes to inflammation, insulin resistance, type 2 diabetes, non-alcoholic fatty liver disease and cardiovascular disease (4).

Mitochondria are organelles found in eukaryotes that are essential for energy metabolism and cellular homeostasis. Mitochondria have unique enzymes and systems that contribute to the citric acid cycle, fatty acid oxidation and oxidative phosphorylation (5). The mitochondria are essential to the differentiation of adipocytes (adipogenesis) and to major adipocyte functions (6). White adipocyte mitochondria are elongated and thin, which are involved in the production of ATP. Mitochondria of brown adipocytes have more quantity and larger body size compared to white adipocytes, and UCP1 located in the inner mitochondrial membrane can cause proton leakage across the inner membrane of the mitochondria, thus transforming the electrochemical energy into heat (7). The pathological expansion of the adipose tissue expansion is accompanied by the downregulation of mitochondrial oxidative pathways and changes in mitochondrial shape and number, ultimately leading to cell death (8–10). Mitochondrial dysfunction has deleterious effects on important adipocyte biology processes (lipid metabolism, adipocyte differentiation, insulin sensitivity, and thermogenesis), leading to metabolic diseases such as obesity and type 2 diabetes (11). Recent studies have shown that mitochondrial function can be improved by adding some herbal extracts and natural compounds in the diet, thereby inducing browning of WAT and adaptive thermogenesis of BAT to maintain metabolic homeostasis.

In this review, we focus on the specific role of mitochondria in adipocytes, summarize the mechanisms of mitochondrial regulation of adipose tissue, and discuss potential therapeutic agents from the perspective of treating obesity, which provide new insights for clinical treatment.

## 2 Research methods

### 2.1 Search strategy

The reporting items were created in strict accordance with the systematic review statement. All articles were retrieved from the PubMed, Embase and Web of Science databases. We used search words such as ‘Mitochondria’, ‘Obesity’, ‘Adipose Tissue, White’, and ‘Adipose Tissue, Brown’ in conjunction with Boolean operators. Within each concept, we combined subject words and free words with the ‘OR’ Boolean operator, and the four concepts were combined with the ‘AND’ Boolean operator. The specific search process can be found in the supplementary materials.

### 2.2 Study selection

**Figure 1:**
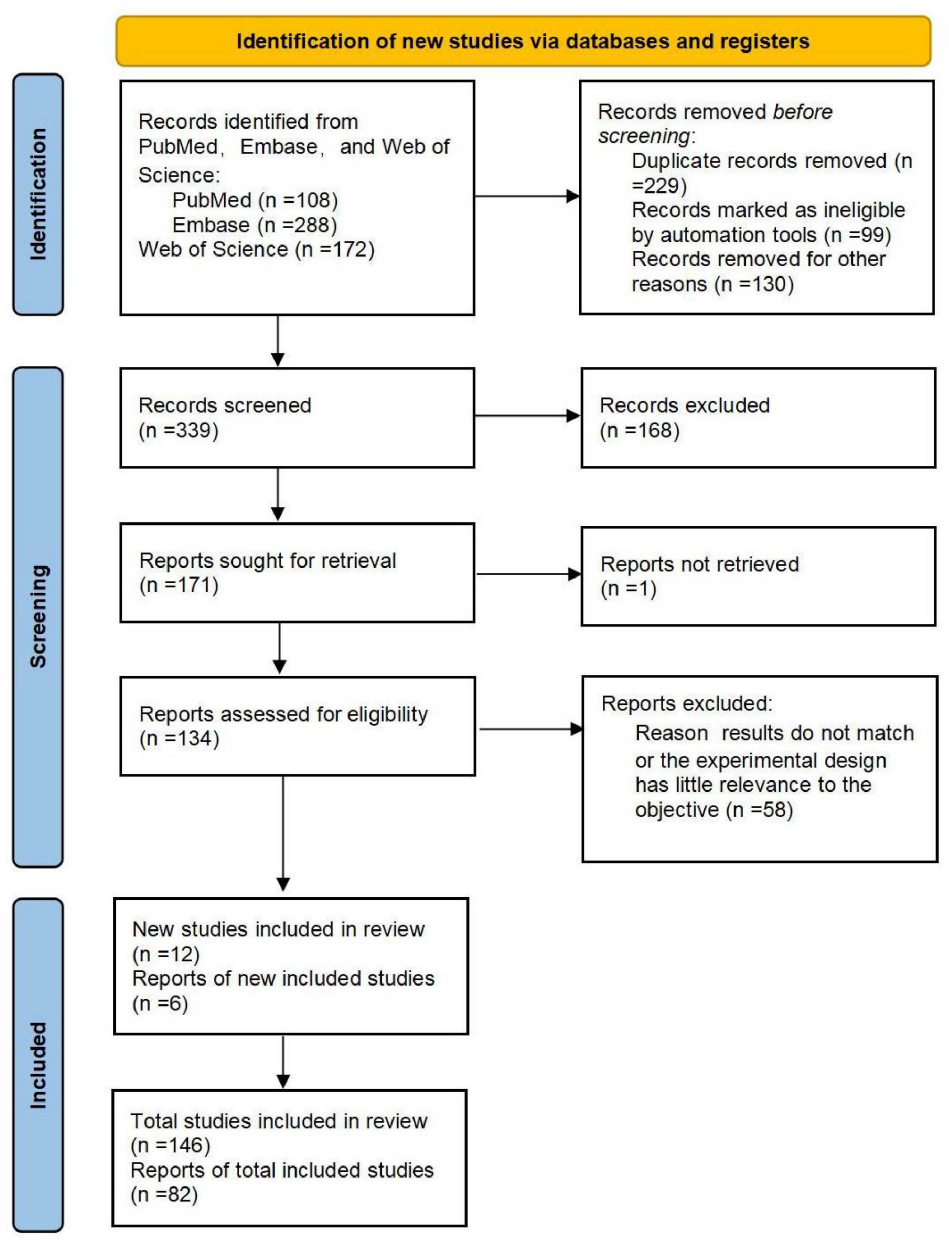
The flow of the literature retrieval.

We screened the titles and abstracts of each paper, and articles that were repeated and irrelevant were removed. The inclusion criteria were the mechanism of mitochondrial regulation of adipose tissue and potential reagents for obesity intervention through the mitochondrial pathway. Articles were excluded according to the following criteria: review articles, editorials, commentaries, conference abstracts, case reports, articles written in other languages, letters to the editor and articles not relevant to the main topic. A ‘PRISMA’ flow chart was used to document the selection process (**Figure 1**) (12).

## 3 Results

### 3.1 Mitochondria regulate adipocyte thermogenesis

Adipose tissue is one of the significant features in maintaining systemic energy homeostasis and insulin sensitivity, not only as a storehouse of excess energy substrates but also as a metabolic health sensor and regulator of energy storage and expenditure. Thermogenesis in adipose tissue is activated in a state of metabolic overload to rapidly utilize excess nutrients. BAT disrupt electron transport in the respiratory chain by activating UCP1 on the mitochondria, thereby preventing the production of ATP and converting energy into heat (13). In addition, mitochondria provide energy for thermogenesis due to efficient proton gradient generation during catabolic processes and electron transport chain (ETC) function (14). It has been reported that individuals with obesity have reduced adipocyte UCP1 expression and that activation of UCP1 improves obesity and metabolic complications. Therefore, targeting mitochondria to activate UCP1 is a strategy to treat obesity (15).

Sirtuin 3 (SIRT3) is a mitochondrially localized deacetylase (16) that belongs to the sirtuin family. Sirtuins are evolutionarily conserved deacetylases whose activity is dependent on NAD+ and have a variety of physiological functions, including the regulation of cell proliferation, DNA repair, antioxidant activity and mitochondrial energy homeostasis. One study reported that caloric restriction activated SIRT3 expression in WAT and BAT, and in BAT from genetically obese mice, SIRT3 was downregulated along with genes related to mitochondrial function, suggesting that SIRT3 activates mitochondrial function and contributes to adaptive thermogenesis in BAT (17, 18). The peroxisome biogenesis factor Pex16 (19), the angiogenic factor VEGF-A (20), disulfide-bond-A oxidoreductase-like protein (DsbA-L) (21), and PR domain-containing 16 (PRDM16) (22) may also regulate thermogenesis by regulating mitochondrial function.

The overexpression of hypoxia-inducible factor-1α (HIF-1α) in adipose tissue inhibits thermogenesis and cellular respiration in BAT and promotes weight gain in mice, which is associated with a reduction in oxygen consumption in BAT, while the decrease in oxygen consumption may be mediated by a reduction in mitochondria (23). Carbohydrate response element-binding protein (ChREBP) is one of the major transcription factors regulating lipogenesis (24). One study showed that overexpression of ChREBP-β reduced the expression of genes involved in mitochondrial biogenesis, autophagy and respiration, leading to a bleached phenotype of BAT (25), which suggested that ChREBP-β is a negative regulator of thermogenesis in BAT. Bone morphogenetic protein 7 (BMP7) has also been shown to upregulate UCP1 and increase adipocyte thermogenesis (26). The absence of the membrane-associated estrogen receptor G protein-coupled receptor 30 (GPR30) may promote BAT mitochondrial uncoupling of respiration (27), suggesting that it is a negative regulator of thermogenesis and contributes to reduced obesity. Optic atrophy protein 1 (OPA1) (28), Brain-derived neurotrophic factor (BDNF) (29), also plays an important role in the adaptive thermogenesis of BAT.

Fat burning relying on adaptive thermogenesis has emerged as a viable strategy to reduce obesity, and the activation of mitochondrial-localized UCP1 and related molecules targeting mitochondria becomes a potential driving force to execute this strategy. Therefore, mitochondria are an essential organelle for maintaining adipocyte metabolic homeostasis.

### 3.2 Activation of WAT browning and BAT whitening

Some WATs exhibit a BAT phenotype when exposed to certain stimuli, which is called “the browning of WAT”. WAT browning produces beige adipocytes, which exhibit UCP1-dependent thermogenesis, and fibroblast growth factor 21 (FGF21) was shown to play an important role in this thermogenesis (30). WAT browning has been found to suppress diet-induced obesity and improve systemic energy metabolism in many animal models (31, 32). Therefore, WAT browning has been investigated as an alternative therapy to BAT thermogenesis. Exercise is a major driver of fat browning. In this sense, the impact of myokines, which are factors secreted by the contracting muscle, on fat browning has provided a molecular mechanism to explain the benefits of exercise on weight loss and metabolic disease prevention. In this sense, several myokines act as positive (FNDC5/irisin, FNDC4, BAIBA and meteorin-like) and negative (myostatin) regulators of fat browning (33–37).

The role of mitochondria in WAT browning cannot be ignored. As an important factor of mitochondrial function, the expression of peroxisome prolilerators-activated receptor γ coactivator l α (PGC1α) was significantly increased in WAT of protein kinase Cβ (PKCβ) deficiency on profound obesity, double knockout (DBKO) mice (38). OPA1, a key protein for mitochondrial fusion, promotes browning of white adipocytes (39).Human white adipocyte mitochondrial activity is regulated by the ubiquitin carrier protein 9/microRNA-30a axis, which is involved in controlling white adipocyte browning (40). MiR-337-3p in adipocytes inhibits Twist1, a negative feedback regulator of BAT metabolism, and enhances adipocyte browning (41). It has also been found that the small molecule compounds RepSox (42), Endonuclease G (EndoG) (43), milk fat globule membrane (MFGM) and its components phosphatidylcholine (PC) (44), bone morphogenetic protein-4 (MBP4) (45), cannabinoid receptor type 1 (CB1R) (46), and E2F transcription factor 1(E2F1) (47) can be involved in the induction of WAT browning. Electroacupuncture (EA) has also been shown to remodel WAT to BAT by deacetylating SIRT-1-dependent peroxisome proliferator‐activated receptor gamma (PPARγ) and regulating the PGC1α-TFAM-UCP1 pathway in order to induce mitochondrial biogenesis (48). These appears to be potential strategies for the treatment of obesity through WAT browning.

The “whitening” of BAT is closely related to obesity-related BAT dysfunction. It is worth mentioning that the process of BAT whitening associated with obesity is reversible by cold exposure and bariatric surgery (49, 50). It is possible that inflammation in whitened BAT contributes to the typical inflammatory state found in obesity (49). Shimizu, I et al. (51) showed that vascular rarefaction results in mitochondrial dysfunction and loss in BAT, which is a noteworthy causal factor in the whitening of BAT in mice models and could affect obesity and obesity-linked diseases. Activation of mTOR signaling downregulated PGC1α, a key activator of mitochondrial biosynthesis, and nuclear respiratory factor 1 (NRF1), an important transcriptional regulator, and downregulated Mitofusin 2 (Mfn2) and OPA1, genes involved in mitochondrial dynamics (52), which may have led to the “whitening” of BAT, suggesting that inhibition of the mTOR signaling pathway is a potential therapeutic avenue for obesity.

**Figure 2:**
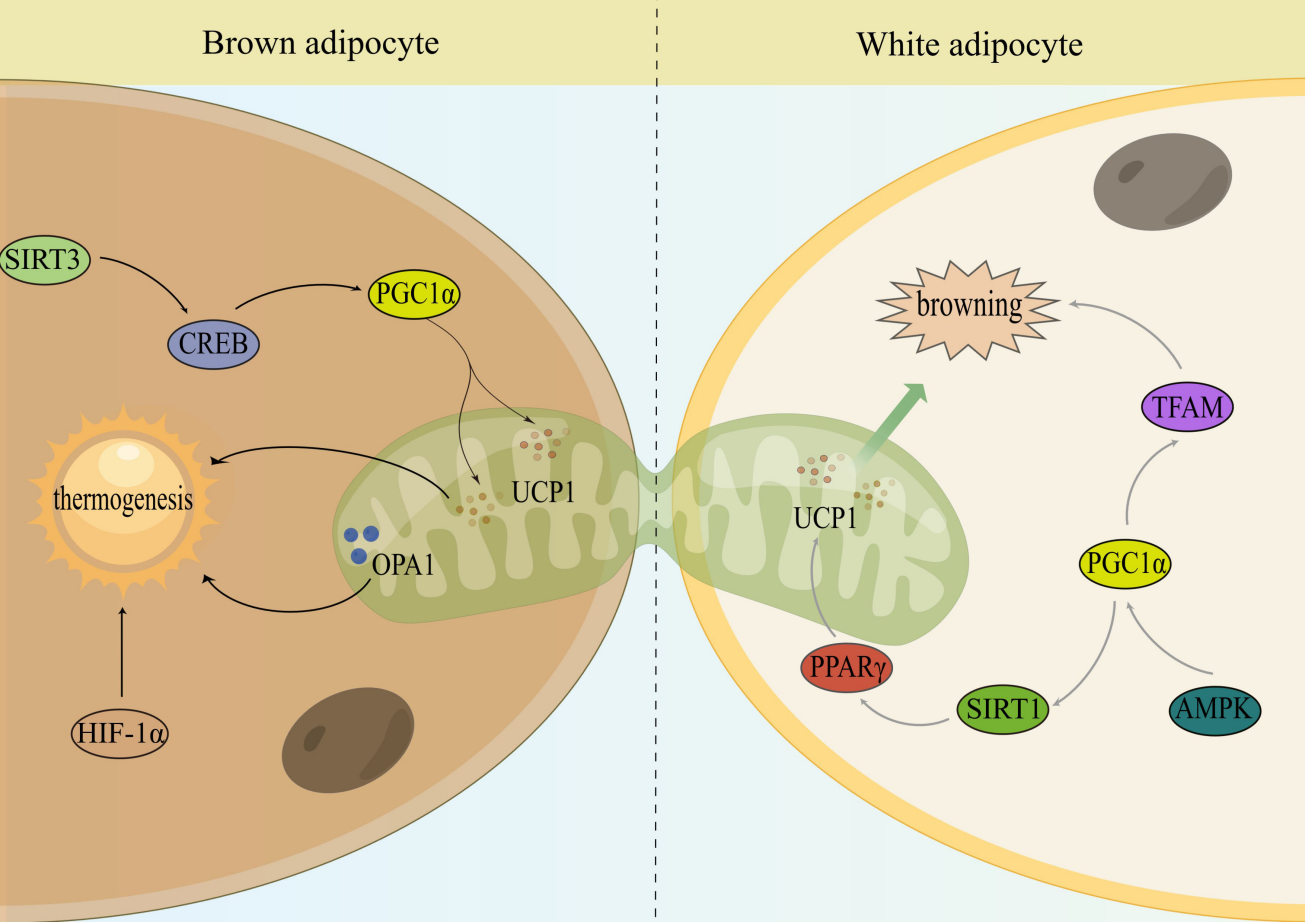
Mitochondria monitor the thermogenesis of brown and activate the browning of WAT. Sirtuin 3 (SIRT3); cAMP response element-binding protein (CREB); peroxisome proliferator-activated receptor-γ cofactor 1α (PGC1α); uncoupling protein-1 (UCP1); optic atrophy 1 (OPA1); hypoxia-inducible factor-1α (HIF-1α); peroxisome proliferator-activated receptor gamma (PPARγ); sirtuin 1 (SIRT1); AMP-activated protein kinase (AMPK); mitochondrial transcription factor A (TFAM).

Obesity is associated with reduced WAT browning and BAT thermogenesis, and some transcriptional regulators and signaling pathways can regulate WAT browning and BAT thermogenesis by improving mitochondrial quality control, suggesting that targeting mitochondrial regulation is an important entry point for obesity prevention and treatment (**Figure 2**).

### 3.3 Mitochondrial regulation of glucolipid metabolism in subjects with obesity

Mitochondrial dynamics in adipocytes may play a key role in initiating systemic metabolic dysregulation. Nutrient overload promotes hypoxia in BAT, which leads to whitening through mitochondrial dysfunction and loss, subsequently leading to impaired systemic glucose metabolism (53). Bean and coworkers (54) identified that the OPA1 gene regulates insulin sensitivity and adipose tissue functions, and controlled OPA1 overexpression in mice could reduce body weight, improve glucose metabolism and insulin sensitivity, reduce fat accumulation and promote browning of white adipocytes. Mfn2 is a gene that promotes mitochondrial fusion and mitochondrial-endoplasmic reticulum interactions. When Mfn2 is knocked down in adipocytes, adult mice consume more food, gain more fat, and have impaired glucose metabolism in standard diets (55). Mfn2 also plays a key role in the regulation of brown adipose tissue thermogenesis favouring mitochondria to lipid droplet interactions (56).

A dysfunctional mitochondrial system in WAT is implicated in obesity-related insulin resistance. OPA1 deletion completely prevented the increase in adiposity and improved insulin fate sensitivity in mice fed a high-fat diet (57). MLX interacting protein-like (MLXIPL) is a transcriptional regulator. MLXIPL-deficient mice are resistant to excessive lipid accumulation and heat-induced mitochondrial degradation in brown adipocytes (58). This result suggested that knockout of MLXPIL may be a potential therapeutic target for obesity-related metabolic diseases. Takaya et al. (59) found that the expression of UCP1 was increased in BAT-derived cultured preadipocytes and their local transplantation reduced inguinal fat pad weight. This finding suggests that local transplantation of BAT-derived preadipocytes may increase energy expenditure and thus reduce obesity. In the tricarboxylic acid cycle, pyruvate is transported to mitochondria by pyruvate carrier 1 (MPC1) to be oxidized to acetyl coenzyme A. Inhibition of MPC1 inhibits pyruvate transport and thus activates fatty acid oxidation (60), this suggests that MPC1 may be an important regulator of mitochondrial energy metabolism.

Mitochondria play a key role in the regulation of glucose utilization and lipid metabolism in adipocytes. By regulating mitochondrial biogenesis and mitochondrial dynamics in adipocytes, the development of obesity and metabolic diseases can be improved.

### 3.4 Other mitochondrial regulation approaches in obesity

Mitophagy is a process that selectively removes damaged mitochondria through a specialized form of autophagy and is essential for mitochondrial quality control (mitochondrial QC) and metabolic homeostasis. The mitochondrial autophagy receptor Fundc1 is a newly defined mitophagy receptor, and mice lacking Fundc1 develop more severe obesity and insulin resistance when fed a high-fat diet (HFD), and disruption of Fundc1 leads to impaired mitophagy and mitochondrial quality in WAT (61). PGC1α is essential for maintaining energy homeostasis. Overexpression of PGC1α in epicardial adipose tissue induces mitosis and improves mitochondrial function, resulting in improved adipose tissue quality (62). The deletion of mitochondrial transcription factor A (TFAM) in adipose tissue results in reduced mtDNA copy number, mitochondrial dysfunction, increased mitochondrial oxidation and positive metabolic effects (63, 64), suggesting that adipose tissue mitochondrial biosynthesis regulation may be a potential therapeutic target for the treatment of obesity.

The expression of PTEN-induced kinase 1 (PINK1), a protein involved in mitochondrial autophagic clearance, was upregulated in the WAT of HFD mice (65). Deletion of PINK1 induced BAT dysfunction, suggesting that the regulation of mitochondrial autophagy contributes to the “whitening” of adipose tissue during the development of obesity (66). The transition from beige to white adipocytes was associated with decreased mitochondria and increased autophagy (67), uncovering a mechanism by which autophagy-mediated mitochondrial clearance controls the maintenance of beige adipocytes, thus providing an opportunity to combat obesity.

Intercellular mitochondrial transfer can support the survival of cells with impaired metabolism. Rohatgi et al. (68) found that adipocytes and macrophages employ intercellular mitochondrial transfer as an immunometabolic crosstalk mechanism to regulate metabolic homeostasis, which is impaired in obesity. Borcherding et al. (69) showed the existence of a potential direct dietary mechanism on the basis of the above studies that could largely ameliorate mitochondrial translocation.

### 3.5 Mitochondrial targeting as a strategy to treat obesity

#### 3.5.1 Herbal extracts and natural compounds

Increasing energy expenditure is a common approach to obesity prevention, and activating BAT may be a potential strategy against obesity. Beta vulgaris has been shown to have an anti-obesity effect by increasing UCP1 during nonshivering thermogenesis in brown adipocytes (70). Liu Z et al. (71) studied the effects of sesamol on disorders of adiposity and fat-related metabolism in mice fed a Western diet. They found that sesamol reduced WAT and BAT mass and adipocyte size by improving the expression of mitochondria-related genes, including PGC1α and UCP1. Jung Y et al. (72) investigated the anti-obesity effects of vanillic acid (VA) *in vivo* and *in vitro* and found that VA increased mitochondrial and thermogenesis-related factors such as UCP1 and PPARγ-1 in BAT and primary cultured brown adipocytes from mice, suggesting the potential of VA as a thermogenesis-activated anti-obesity agent.

Stimulating browning of white adipose cells helps to limit obesity and related metabolic disorders. Lactobacillus plantarum dy-1 (LFBE) can suppress obesity by enhancing thermogenic processes in BAT and browning of adipose tissue in the epithelium using a UCP1-dependent mechanism of activation (73). Averrhoa bilimbi can also induce adipocyte browning and enhance mitochondrial activity due to upregulation of UCP1 (74). Kang J et al. (75) studied the effects of secoisolariciresinol diglucoside (SDG) on WAT browning and found that SDG increased UCP1, PGC1α and PRDM16 in WAT and BAT in mice, as well as mitochondrial biogenesis and activation, suggesting that SDG is a potential candidate for ameliorating obesity and other metabolic disorders. Lycopene (LYC), one of the major carotenoids in tomatoes, has been used preclinically and clinically in the treatment of obesity and type 2 diabetes. LYC can induce browning and enhance mitochondrial respiration in white adipocytes and improve glucose and lipid metabolism by upregulating PPARγ (76).

The whitening of BAT during obesity and aging promotes metabolic disorders and related diseases. Gao P et al. (77) determined that the inhibitory effect of capsaicin on HFD-induced obesity and BAT whitening was dependent on the involvement of SIRT3, which could mediate the beneficial effects of capsaicin on attenuating reactive oxygen species production, increasing mitochondrial activity and limiting HFD-induced mitochondrial calcium overload.

Regulation of mitochondrial biogenesis is also a potential avenue for the treatment of obesity. The ethanolic extracts of both rutin and Platycodon grandiflorum (PG) can provide benefits for obesity by increasing the expression of genes involved in mitochondrial biogenesis (78, 79). α-Lipoic acid (α-LIP) is a naturally occurring antioxidant that promotes mitochondrial biogenesis and brown-like remodeling in cultured white subcutaneous adipocytes from donors with obesity (80). Marqués and colleagues (81) found that resveratrol has potential therapeutic effects in improving mitochondrial biogenesis (**Table 1**).

**Table 1:** Key factors in herbal extracts and natural compounds.

Name	Key factors	Function	Reference
**Beta vulgaris**	*UCP1, PPARγ*	Adaptive thermogenesis	(70)
**VA**	UCP1*, PPARγ-1α, PPARγ, C/EBPα, AMPKα*	Adaptive thermogenesis	(72)
**LFBE**	*UCP1, PPARγ, C/EBPα, SREBP-1c, aP2, Cidea*	Adaptive thermogenesis	(73)
**Averrhoa bilimbi**	*UCP1, PPARγ*, PRDM16	WAT browning	(74)
**SDG**	UCP1, PGC1α, PRDM16, AMPK*α*	WAT browning and mitochondrial biogenesis	(75)
**LYC**	*UCP1, PPARγ, PPARγ-1α*, PRDM16	WAT browning	(76)
**Capsaicin**	SIRT3, AMPK, MCU, H3K27ac	Mitochondrial biogenesis	(77)
**Rutin**	UCP2, PGC1α*, PPARγ, SREBP-1c, aP2*	Mitochondrial biogenesis	(78)
**PG**	UCP1, PGC1α*, PPARγ*, PRDM16, SIRT3, NRF, Cyto C, SIRT1*, C/EBPα, AMPKα*	Mitochondrial biogenesis	(79)
**α-Lip**	PGC1α, SIRT1, PRDM16, NRF1, TFAM, Cidea, Tbx1	WAT browning and mitochondrial biogenesis	(80)
**Resveratrol**	UCP1*, PPARγ*, PRDM16, Cidea, CD137, TMEM26, COX4α, NRF, TFAM, IGFBP3	Mitochondrial biogenesis and WAT browning	(81) (82)
**Sesamol**	UCP1, PGC1α	Mitochondrial biogenesis	(83)
**Phytol**	UCP1, PGC1α, Cyto C, Cidea, PRDM16, AMPK*α, CD137, TMEM26*	Adipocyte differentiation	(84)
**Ursodeoxycholic Acid**	PGC1α, STAT3, CD36, SREBP-1	WAT browning and mitochondrial biogenesis	(85)
**Multi-Ingredient Supplement**	UCP1, PGC1α*, PRDM16*, *Cidea*	WAT browning and mitochondrial biogenesis	(86)
**T3**	UCP1, PGC1α*, PRDM16*, TOMM20, VDAC1	WAT browning and mitochondrial autophagy	(87)

Some compounds can also activate mitochondrial biogenesis in skeletal muscle. Resveratrol was shown in 2006 to affect skeletal muscle mitochondrial biogenesis and thus metabolic homeostasis by decreasing PGC1α acetylation and increasing PGC1α activity (88). As a thermogenic tissue, skeletal muscle is also capable of regulating energy expenditure. When UCP1 is absent or non-shivering thermogenesis is affected, skeletal muscle generates heat through shivering and is supplied with energy by carbohydrates and lipids (89). Thermogenesis in skeletal muscle is dependent on the homologue of UCP1, uncoupling protein 3 (UCP3). 5,3’-Triiodo-L-thyronine (T3) was reported to induce UCP3 expression to regulate skeletal muscle thermogenesis (90). It is interesting to note that these compounds also work through similar pathways in BAT. Recruitment of BAT and skeletal muscle bear very high degree of similarities (91). Both organs are highly vascularized, neuralized, and contain abundant mitochondria (92). Studies by Bal et al. (93) have shown that skeletal muscle thermogenesis can be activated to compensate for the absence of BAT, suggesting that the two thermogenic systems can be functionally complementary. It has been shown that PRDM16 can control the bidirectional transformation of brown adipocytes and skeletal muscle cells (94), which may represent two different potential therapeutic targets to expand the thermogenic capacity of adipose tissue.

#### 3.5.2 Traditional Chinese medicine and other alternative medicines

Zhang et al. (95) found that the natural antioxidant Lycium could enhance UCP1 expression, upregulate PGC1α and induce browning in white adipocytes, as well as enhance glucose uptake and oxidative utilization, lipolysis and fatty acid oxidation in 3T3-L1 adipocytes, and the application of Lycium is a promising strategy to combat obesity and obesity-related metabolic disorders. Huangqi San (HQS) is a traditional Chinese medicine formula, and Hao M et al. (96) found that it could significantly increase the number of mitochondria, increase the expression of UCP1 and PGC1α in BAT, and improve metabolic disorders and lipid deposition in hyperlipidemia in obese rats after 13 W intravenous injection of HQS, suggesting that HSQ is an effective drug for the treatment of hyperlipidemia with obesity. Tanshinone IIA (TAN2A) is a major active ingredient of the traditional Chinese medicine tanshinone, and tanshinone 20 (TAN20) is a derivative of TAN2A. Ma L et al. (97) showed that both TAN2A and TAN20 were able to increase mitochondrial content in adipose tissue, increase energy expenditure and reduce body weight, thereby improving insulin sensitivity and metabolic homeostasis in mouse models of obesity and diabetes. It has also been shown (98) that administration of tanshinone 1 (TAN1) prevents HFD-induced obesity in mice, which was associated with enhanced expression of brown adipocyte-related genes in WAT and BAT, and that TAN1 also led to increased mtDNA content and lipolysis.

Drugs with therapeutic potential can combat obesity by affecting the differentiation of white adipocytes. Ravaud C et al. (99) showed that HIV protease inhibitors (PIs) can reduce the expression of UCP1, improve mitochondrial function in brown adipocytes and regulate thermogenesis and are promising potential drugs against obesity. Empagliflozin can increase mitochondrial biogenesis and fusion, improve their function and promote browning of 3T3-L1 adipocytes (100), FAM134B improves adipocyte differentiation by enhancing mitophagy (101), and melatonin drives WAT browning (102), which are also promising anti-obesity treatments.

Targeting mitochondria as a therapeutic strategy for metabolic diseases is being extensively investigated as a new potential approach. In conclusion, the possibility of reducing obesity and metabolic diseases by mediating mitochondrial biogenesis or mitochondrial dynamics to increase thermogenesis, activate WAT brownout, and promote lipid metabolism and fatty acid oxidation is being progressively demonstrated.

### 3.6 Conclusion

The importance of mitochondria in energy metabolism and cellular homeostasis has been extensively noted and studied. Since the rediscovery of active brown and beige adipocytes in humans a decade ago, more and more research has focused on the regulation of adipocyte function by mitochondria. We summarized the searched articles and showed that mitochondria are involved in regulating the thermogenesis of BAT and increasing energy expenditure; in lipid homeostasis of adipose tissue and regulating glucose metabolism; and are also able to participate in inducing browning of WAT while promoting the whitening of BAT. These are key therapeutic targets for obesity-related metabolic diseases. The regulation of mitochondrial biogenesis, mitochondrial autophagy and mitochondrial translocation are also potential therapeutic opportunities for obesity. In fact, a series of studies are working on the interpretation of drugs or potential drugs for the treatment of obesity through these mitochondrial pathways.

In conclusion, mitochondria-targeted drugs for adipocytes are very promising. Therefore, understanding the molecular mechanisms underlying adipocyte mitochondrial dysfunction and the pathogenesis of obesity-related metabolic diseases is crucial for the development of new therapeutic approaches. However, future studies need to elucidate the mechanisms by which mitochondria undergo metabolic disorders to better account for their pathogenic role in metabolic diseases.

## Data availability statement

The original contributions presented in the study are included in the article/**Supplementary Material**. further inquiries can be directed to the corresponding authors.

## Author contributions

YZ and HongyaY conceived the research question. YZ wrote the first draft of the manuscript with the support of WD, YP and YG. HongY and NY discussed the results. All authors contributed to the article and approved the submitted version.
