# Supplementary material for: Mitochondria-associated regulation in adipose tissues and potential reagents for obesity intervention
Source: Front Endocrinol (Lausanne). 2023 Jun 16;14:1132342. doi: 10.3389/fendo.2023.1132342 (PMC10313115; doi:10.3389/fendo.2023.1132342)
Supplement: Supplementary file 1 [file DataSheet_1.pdf]

Supplementary Material

Mitochondria-associated Regulation in Adipose Tissues and Potential Reagents for Obesity Intervention

Yali Zheng*, Weijun Ding, Hongya Yang

*** Correspondence:** Hongya Yang: yhy6018@163.com

1 Supplementary Data

Research formula:

PubMed：#5

Search: (((#1) AND (#2)) AND (#3)) AND (#4)   108

#4

Search: (((((Adipose Tissue, Brown[Title/Abstract]) OR (Brown Fat[Title/Abstract])) OR (Tissue, Brown Adipose[Title/Abstract])) OR (Brown Adipose Tissue[Title/Abstract])) OR (Fat, Brown[Title/Abstract])) OR (Hibernating Gland[Title/Abstract])   11,408

#3

Search: ((((Adipose Tissue White[Title/Abstract]) OR (Tissue, White Adipose[Title/Abstract])) OR (White Fat[Title/Abstract])) OR (Fat, White[Title/Abstract])) OR (White Adipose Tissue[Title/Abstract])   10,043

#2

Search: Obesity[Title/Abstract]   296,334

#1

Search: (((((mitochondria[Title/Abstract]) OR (Mitochondrion[Title/Abstract])) OR (Mitochondrial Contraction[Title/Abstract])) OR (Contraction, Mitochondrial[Title/Abstract])) OR (Contractions, Mitochondrial[Title/Abstract])) OR (Mitochondrial Contractions[Title/Abstract])

156,305   08:12:32

Embase：

#1 AND #2 AND #3 AND #4   288

#4

'adipose tissue, brown'/exp OR 'adipose tissue, brown' OR (adipose AND ('tissue,'/exp OR tissue,) AND brown) OR 'brown adipose tissue':ab,ti 20,615

#3

'adipose tissue white'/exp OR 'adipose tissue white' OR (adipose AND ('tissue'/exp OR tissue) AND ('white'/exp OR white)) OR 'white adipose tissue':ab,ti 21,797

#2

'obesity'/exp OR obesity OR 'abdominal obesity':ab,ti OR 'adolescent obesity':ab,ti 722,141

#1

mitochondrion:ab,ti OR mitochondria:ab,ti 172,023

web of sciences：

#41 AND #42 AND #43 AND #44 172

(((((AB=(mitochondria)) OR AB=(Mitochondrion)) OR AB=(Mitochondrial Contraction)) OR AB=(Contractions, Mitochondrial)) OR AB=(Mitochondrial Contractions)) OR AB=(Contraction, Mitochondrial) 186,818

(((((AB=(Adipose Tissue, Brown)) OR AB=(Brown Fat)) OR AB=(Tissue, Brown Adipose)) OR AB=(Brown Adipose Tissue)) OR AB=(Fat, Brown)) OR AB=(Hibernating Gland) 21,800

((((AB=(Adipose Tissue White)) OR AB=(Tissue, White Adipose)) OR AB=(White Fat)) OR AB=(Fat, White)) OR AB=(White Adipose Tissue) 39,800

AB=(Obesity) 366,756
